# *IRX3* plays an important role in the pathogenesis of metabolic-associated fatty liver disease by regulating hepatic lipid metabolism

**DOI:** 10.3389/fendo.2022.895593

**Published:** 2022-07-26

**Authors:** Yongqiang Ma, Guangshun Chen, Junfang Yi, Qiang Li, Zhi Tan, Wenling Fan, Xiaohua Luo, Zhiyong He, Zhongzhou Si, Jiequn Li

**Affiliations:** ^1^ Department of Liver Transplant, The Second Xiangya Hospital of Central South University, Changsha, China; ^2^ National Clinical Research Center for Metabolic Diseases, The Second Xiangya Hospital of Central South University, Changsha, China; ^3^ Transplant Medical Research Center, The Second Xiangya Hospital, Central South University, Changsha, China; ^4^ Department of Gastroenterology, The First Hospital of Changsha, Changsha, China

**Keywords:** MAFLD, differentially expressed genes, *IRX3*, lipid metabolism, mitochondrion

## Abstract

Metabolic-associated fatty liver disease (MAFLD) affects approximately a quarter of the global population. Identification of the key genes and pathways involved in hepatic lipid metabolism is of the utmost importance for the diagnosis, treatment, and prevention of MAFLD. In this study, differentially expressed genes were identified through whole-genome transcriptional analysis of liver tissue from MAFLD patients and healthy controls, and a series of lipid metabolism-related molecules and pathways were obtained through pathway analysis. Subsequently, we focused on Iroquois homeobox protein 3 (*IRX3*), one of 13 transcription factors that were screened from the 331 differentially expressed genes. The transcription factor *IRX3* was significantly decreased in the liver tissue of patients with MAFLD when compared with healthy controls. Pearson’s correlation analysis showed that the expression levels of *IRX3* in liver tissue were negatively correlated with serum total cholesterol, triglycerides, low-density lipoprotein cholesterol, and uric acid levels. The overexpression and interference of IRX3 induced the increased and decreased lipid droplet accumulation *in vitro*, respectively. Moreover, interference of *IRX3* expression increased mitochondrial fragmentation and reduced the activity of the mitochondrial respiratory chain complex IV. In summary, the study demonstrated that *IRX3* regulated hepatic lipid metabolism of MAFLD, and also revealed the effect of *IRX3* on mitochondria might be an important mechanism by which *IRX3* regulated hepatic lipid metabolism of MAFLD.

## Introduction

Metabolic-associated fatty liver disease (MAFLD), previously known as non-alcoholic fatty liver disease. has become a worldwide public health problem in recent decades (1, 2). Owing to the large economic and social burden of MAFLD and its complications, developing effective treatments is urgently needed. Although the epidemiology and pathogenesis of MAFLD are well understood, no specific drug treatment has been approved for MAFLD to date (3). Lipid accumulation in hepatocytes is the hallmark of MAFLD initiation, and lipotoxicity in the liver is a key factor in the development of MAFLD (4). Steatosis, a typical characteristic of MAFLD, is expected to be caused by disordered hepatic lipid metabolism (5, 6).

Hepatic lipid homeostasis is a complex regulatory mechanism, resulting from the interaction of multiple genetic and environmental factors (7). Previous studies have shown that *PNPLA3-I148M*, *MBOAT7*, *GCKR*, *TM6SF2-E167K* and *HSD17B13* were closely correlated with liver histological severity of MAFLD (8–12). In addition, many gene variations have been shown to contribute to MAFLD progressions, such as lipid metabolism regulation (*PPARα/γ*, *CD36*, *BHMT2*, *LYPLAL1*, *APOB*, *MTP*, *LPIN1*, *UCP2*), innate immunity (*IL28B*, *MERTK*), insulin signaling pathway (*ENPP1*, *IRS1*), oxidative stress (*SOD2*) and fibrogenesis (*KLF6*) (13). At the same time, epigenetic changes were involved in the occurrence and development of MAFLD, which was manifested in DNA methylation, microRNA (miRNA), histone modification, etc (14). MiRNA regulated the expression of multiple genes by inhibiting or interfering with the stability of RNA. Studies have shown that miR-122, miR-34, and miR-141 were associated with the occurrence of MAFLD (15–17). Consequently, identifying the genes and pathways of hepatic lipid metabolism disorder might provide new targets for the treatment of MAFLD.

In the present study, whole-genome transcriptional analysis of liver tissues from MAFLD patients and controls identified 331 differentially expressed genes, and pathway analysis of these genes revealed a range of molecules and pathways involved in MAFLD. Given the importance of transcription pathways in lipid metabolism, we screened 13 transcription factors from differentially expressed genes, among which Iroquois homeobox protein 3 (*IRX3*) was one of the candidate genes. *IRX3* is a nuclear-distributed gene, studies have shown that the up-regulation of *IRX3* was significantly associated with obesity in humans, and *IRX3* knockout mice significantly enhanced basal metabolism, reduced fat content and ultimately reduced body weight, suggesting that *IRX3* played an important role in regulating body weight and energy metabolism (18, 19). Studies have also showed that the expression level of *IRX3* was closely related to the expression of uncoupling protein 1 (*UCP1*) in brown adipose tissue, thus mediating heat production in adipose tissue (20). All of these studies indicated that *IRX3* played an important role in the lipid metabolism of adipocytes. However, the role of *IRX3* in the hepatic lipid metabolism of MAFLD has not been reported. In the present study, we aimed to clarify the regulatory roles and underlying mechanisms of *IRX3* in the lipid metabolism of MAFLD.

## Materials and methods

### Liver samples

7 patients with histologically proven MAFLD and 9 healthy control samples were included. Control samples were obtained from hepatic hemangioma resection. Three pathologists evaluated the samples, and patients with a non-alcoholic fatty liver disease activity score (NAS) ≥5 were considered to have steatohepatitis (21). All samples were investigated for other possible interfering liver diseases (viral infections, toxic exposure and autoimmune disorders, et al.) and the samples with other concomitant liver diseases were excluded from the study. All experimental participants provided informed consent. The experiments received ethical approval from the ethics committee of the Second Xiangya Hospital of Central South University. The clinical characteristics of the MAFLD patients and controls are shown in **Table S1**. To exclude the effect of estrogen on liver lipid metabolism (22, 23), all 16 patients were male. The mean age of MAFLD patients was 44.86 ± 5.84 years and the mean body mass index (BMI) was 29.04 ± 5.40. The mean age of the healthy controls was 44.56 ± 9.63 years and the mean BMI was 22.68 ± 2.65.

### Serum uric acid and lipids measurements

Blood samples were obtained from the patients and healthy controls. The serum was separated by centrifugation at 3,000 rpm for 15 min to analyze biochemical and hematological parameters. Total cholesterol (TC), triglycerides (TG), alanine aminotransferase, gamma-glutamyl transferase, low-density lipoprotein (LDL-C), and uric acid (UA) selected the first blood drawing results after hospitalization as the target data. The serum was processed in Vitros-250 automated analyzer using readymade dry chemistry kits procured from Ortho-Clinical Diagnostics, Johnson & Johnson, USA. Samples giving readings above or below two SD were reprocessed or discarded.

### Hepatic transcriptome

Total RNA was isolated from liver tissues using the Trizol method following the reagent protocol (Invitrogen, USA). Total RNA was quantified and qualified as previously described (24). Next-generation sequencing was performed using the Illumina sequencing platform at Genergy Bio-Technology Inc. Quality control of the raw fastq files was performed using the software took FastQC v0.11.3. Sequencing reads were trimmed with Trimmomatic v0.36 and aligned to the human genome (GRCh37.p13) using the STAR aligner v2.5.3a (25). Read quantification was performed with RSEM v 1.3.0 (26) and the Gencode release 19 (27).

Differentially expressed genes from RNA-seq data were statistically analyzed. Quantification of gene expression by RNA-seq is to count the number of reads that have mapped to each gene. Read quantification was performed with RSEM v 1.3.0 and the Gencode release 19. Adjusted *p* values for multiple comparisons were calculated applying the Benjamini-Hochberg correction. The events were defined as significant using a cutoff of adjusted *p* value <0.05. Principal component analysis, Gene Ontology (GO), and Kyoto Encyclopedia of Genes and Genomes (KEGG) pathway enrichment analysis, correlation analysis, and hierarchical clustering analysis were performed using R or the Python environment.

### Immunohistochemistry

Liver tissues were fixed with 4% paraformaldehyde, embedded in paraffin. An *IRX3* antibody (Abcam, USA) was used to quantify *IRX3* protein expression using paraffin-embedded sections (5 µm) from each tissue. The sections were incubated as previously described (28). Haematoxylin and eosin (HE) staining and Oil Red O for lipid droplets staining were conducted for histological examination. All stained sections were scanned under a light microscope (Leica, USA). Quantitative analysis was performed using ImageJ software (NIH, USA).

### Transfection

HepG2 and L02 cells were obtained from ATCC and the Chinese Academy of Science (Shanghai, China), respectively. The cells were grown at 37°C with 5% CO_2_ in DMEM (Invitrogen) supplemented with 10% fetal bovine serum, 2 mm l-glutamine, 100 units/ml penicillin and 100 μg/mL streptomycin. The plasmid pcDNA3.1-*IRX3*-Flag was introduced into L02 cells or HepG2 cells *via* transfection with Lipofectamine 2000 reagent (Invitrogen) according to the manufacturer’s protocol.

### RNA interference

Cells were plated the day before transfection at 2*10^4^ cells per well in 12-well plates. Oligos complementary to *IRX3* RNA and non-target oligos were synthesized by GenePharma (Sangon Biotech, China). The target sequence of *IRX3* siRNA is as follow: 5’-CACTGACGAGGAGGGAAACGCTTAT-3’. The oligos for *IRX3* interference were transfected into HepG2 cells using the Lipofectamine 2000 reagent kit (Invitrogen) according to the manufacturer’s protocol. The human primer sequences for *IRX3* were as follows: 5′-CTCTCCCTGCTGGGCTCT-3′ (forward) and 5′-CAAGGCACTACAGCGATCTG-3′ (reverse).

### BODIPY and immunofluorescence staining

Cells were grown on glass coverslips. After transfection for 48 h, the cells were treated with palmitic acid and oleic acid and detected as previously described (24). Cells were fixed in 4% formaldehyde for 15 min, permeabilized, and blocked with 0.1% Triton X-100 in 4% appropriate normal serum in PBS for 1 h. Immunofluorescence staining was performed using primary antibodies against TOM20 for mitochondrial staining Flag for exogenous expression of IRX3. The fluorescent dye BODIPY 493/503 (2.0 µm; D3922, Invitrogen) was used for lipid droplet staining. Finally, the nuclei were stained with 4′,6-diamidino-2-phenylindole (DAPI; Invitrogen). Cells were imaged using a Zeiss LSM880 confocal microscope using an X63 Plan Apo objective (Zeiss). The lipid content can be quantified as the number of green spots per cell. Mitochondrial morphology was quantified from auto-segmented images with an ImageJ macro reporting several measures including form factor (FF = (perimeter^2^)/(4 *π* area)), aspect ratio (AR = major axis/minor axis), and length as previously described (29).

### Isolation of mitochondria and measurement of mitochondrial complexes and citrate synthase activity

Cells were washed twice with phosphate-buffered saline and incubated for 30 min on ice in lysis buffer (68 mM sucrose, 200 mM mannitol, 50 mM potassium chloride, 1 mM ethylenediaminetetraacetic acid (EDTA), 1 mM EGTA, and 1 mM dithiothreitol with a protease inhibitor cocktail. The cells were then lysed using 45 passages through a 25G 5/8 needle and centrifuged at 1,500 g for 10 min. Cytosolic extracts were recovered after centrifugation at 13,000 g for 20 min. The pellet contained the mitochondria. The total protein concentration was determined using the BCA kit (Pierce, USA) according to the manufacturer’s instructions. The mitochondrial activity of complex I (ab109903, Abcam), complex II + III (b109905, Abcam), complex IV (ab109906, Abcam), and complex V (ab109907, Abcam) was determined using *in vitro* assays following the manufacturer’s procedures. The citrate synthase activity of mitochondrial extracts was measured using a Citrate Synthase Activity Assay Kit (ab119692, Abcam) according to the manufacturer’s instructions.

### Data and statistical analysis

Metascape online software was used for pathway enrichment analysis. Protein-protein interaction (PPI) enrichment was performed using STRING and Metascape. The molecular complex detection (MCODE) algorithm, pathway, and process enrichment analysis were applied to identify the densely connected components of the network using Metascape. The three best-scoring terms defined by the *p*-value were selected as the functional description of the corresponding components. Data are presented as means ± standard error using Prism 8.3.0 software. Statistical analysis of the data of RNA-seq and *in vitro* experiments was performed using an unpaired 2-tailed *t*-test with 95% confidence interval in Prism.

## Results

### Transcriptome analysis revealed a series of novel genes and pathways involved in MAFLD

**Figure 1:**
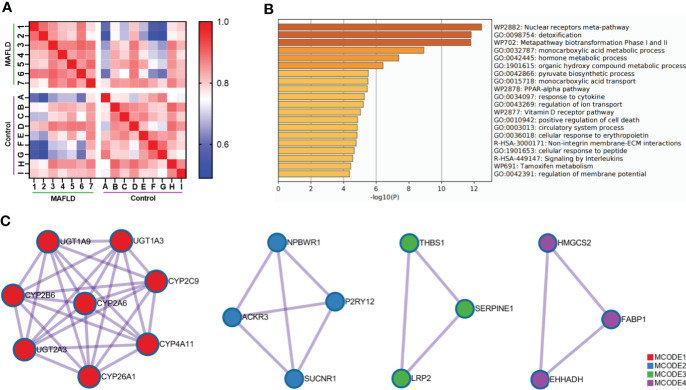
Comprehensive analysis of differentially expressed genes in liver tissue between MAFLD patients and controls. **(A)** Pearson’s correlation heat map of 331 differentially expressed genes. The darker blue color indicated a lower correlation and greater variability between the two individuals. **(B)** Bar graph of the top 20 enriched biological processes and pathways related to MAFLD. **(C)** The four significant MCODE components from the PPI network. The dots represented proteins, and the lines between the dots represented interactions between proteins.

To identify differentially expressed genes between patients with MAFLD and healthy controls, transcriptome analysis was performed on liver tissue samples. A total of 331 differentially expressed genes were identified (**Table S2**, *p*<0.05, log2 (fold change) ≥1.0 or ≤ −1.0). The Pearson’s correlation heat map revealed significant differentially expressed genes between the two groups (**Figure 1A**). To further study the function of differentially expressed genes, GO and KEGG pathway enrichment analysis was performed using Metascape (**Table S3**). The top 20 significantly enriched biological processes were listed in **Figure 1B**. Of note, these genes were significantly enriched in the nuclear receptors meta-pathway, monocarboxylic acid metabolic process, pyruvate biosynthetic process, *PPAR* signaling pathway and organic hydroxy compound metabolic process pathways. Then, PPI network analysis was used to further explore protein interactions between the differentially expressed genes. **Figure 1C** showed four important MCODE components from the PPI network by Metascape. Finally, through the pathway analysis of each MCODE component, the representative role of each MCODE component was obtained, including biological oxidation, lipid metabolism, *PPAR* signaling pathway, blood vessel morphogenesis and retinol metabolism (**Table S4**). Through the analysis of differentially expressed genes, we found that many of these biological functions or processes are directly or indirectly associated with lipid metabolism.

### Expression of IRX3 was decreased in the liver tissue of MAFLD patients

**Figure 2:**
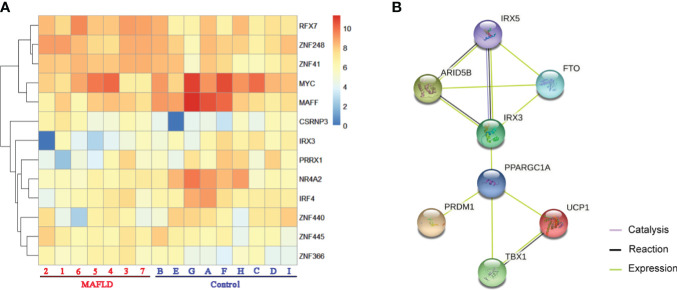
The transcription factors were screened from differentially expressed genes. **(A)** Heatmap of transcription factors from all samples, the darker of orange color indicated the higher the gene expression. **(B)** Action view of genes related to the FTO obesity variant mechanism pathway. The colors corresponded to interactions according to the legend (bottom right).

Among these differentially expressed genes, transcription factors such as *MYC* (MYC proto-oncogene, BHLH transcription factor), *IRF4* (Interferon regulatory factor 4), and *MAFF* (MAF bZIP transcription factor F) were identified, which have been demonstrated to regulate lipid metabolism (30–32). A total of 13 transcription factors were screened from the 331 differentially expressed genes (**Table S5**) and differences in the expression of these transcription factors were shown in the cluster analysis heat map (**Figure 2A**).

**Figure 3:**
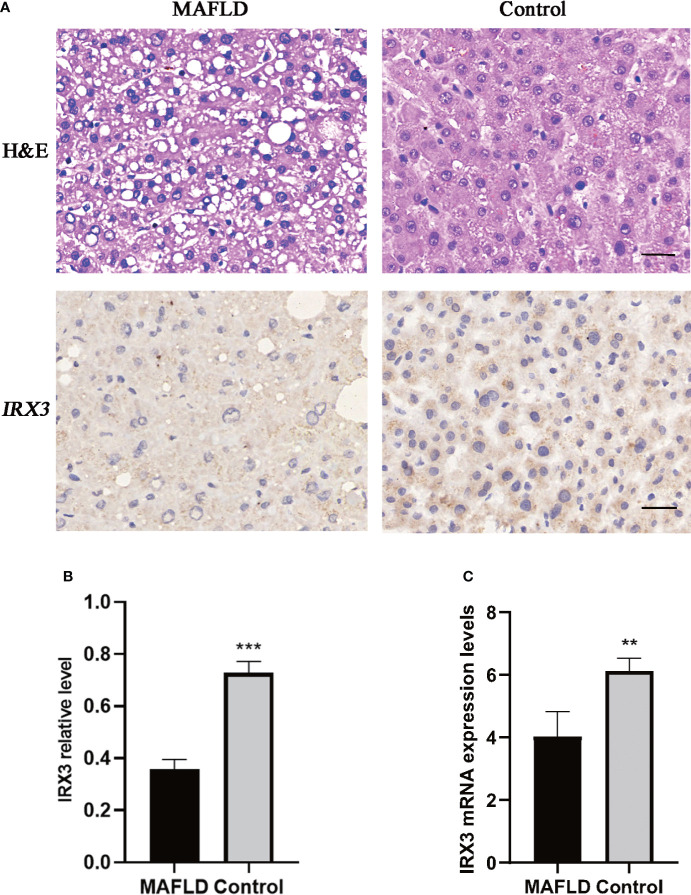
The expression of *IRX3* was decreased in the liver tissue of MAFLD patients. **(A)** Hemotoxylin and eosin (H&E) staining and immunohistochemistry assay for *IRX3* expression in liver tissues of MAFLD patients and controls. **(B)** Statistical results of the relative expression of *IRX3* in liver tissue samples by immunohistochemical staining (n=7, 2–3 slides/patient, ***p<0.001). **(C)** The mRNA expression levels of *IRX3* in liver tissue samples (**p<0.01).

*IRX3*, one of these 13 transcription factors, has been demonstrated to play an important role in the occurrence and development of obesity by participating in the fat mass and obesity associated (*FTO*) obesity variant mechanism (33–35) (**Figure 2B**). However, the role of *IRX3* in hepatic lipid metabolism has not been reported. Immunohistochemical staining showed that the expression of *IRX3* was decreased in the liver tissue of MAFLD patients compared with controls (**Figures 3A, B** **)**. The RNA-seq results for the liver tissue samples also confirmed the lower expression of *IRX3* in MAFLD (**Figure 3C**, *p*<0.01). These results suggested that *IRX3* might be involved in the pathogenesis of MAFLD.

### *IRX3* was highly correlated with the clinical characteristics of lipid metabolism of MAFLD

**Figure 4:**
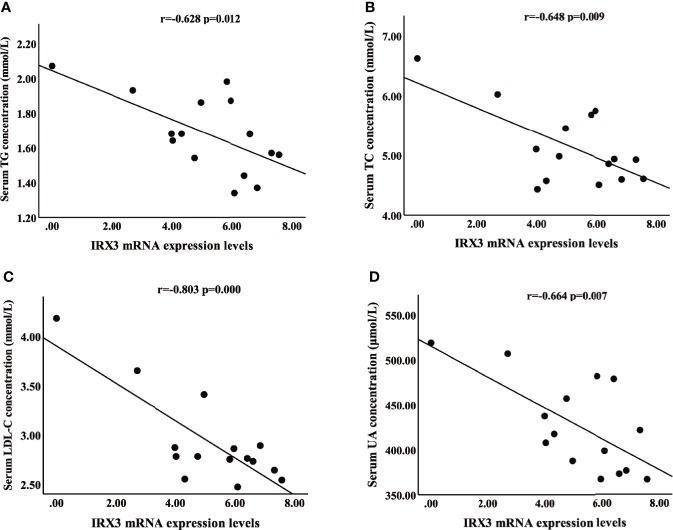
Pearson’s correlation analysis between *IRX3* expression levels and clinical characteristics of lipid metabolism. **(A–D)** Correlation analysis between *IRX3* expression and serum TG, TC, LDL-C and UA levels. TC, total cholesterol; TG, triglycerides; LDL-C, low-density lipoprotein cholesterol; UA, uric acid.

We also analyzed the correlation between the expression level of *IRX3* and the clinical characteristics of lipid metabolism. Pearson’s correlation analysis showed that the expression level of *IRX3* in liver tissue was negatively correlated with serum concentrations of TC (r=-0.648, *p*=0.009), TG (r=-0.628, *p*=0.012), LDL-C (r=-0.803, *p*=0.000) and UA (r=-0.664, *p*=0.007) (**Figures 4A–D**). These results suggested that hepatic *IRX3* might be closely related to the lipid metabolism of MAFLD patients.

### *IRX3* regulated hepatocyte LD accumulation *in vitro*

**Figure 5:**
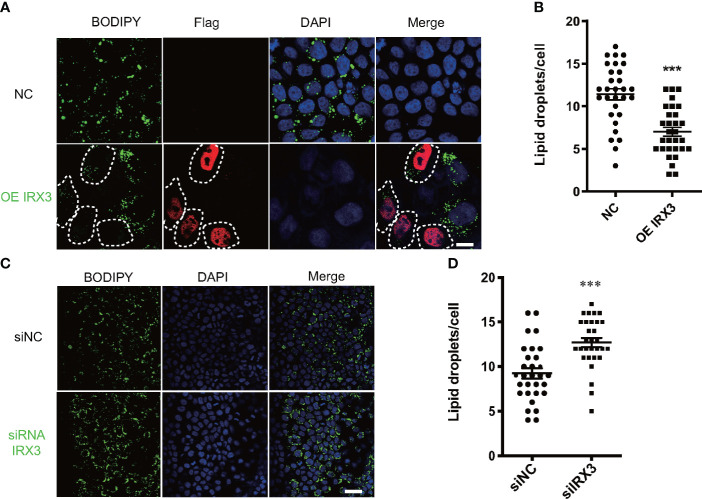
The effect of *IRX3* on lipid droplet accumulation *in vitro*. **(A)** HepG2 cells were transfected with the *IRX3*-Flag (OE *IRX3*, Red) and vector plasmid (NC). IRX3-Flag-positive cells were marked by white dashed boxes. **(B)** The number of lipid droplets per cell shown in A was quantified. **(C)** HepG2 cells were transfected with *IRX3* siRNA and control siRNA. **(D)** Quantification of the number of lipid droplets per cell in **(C)** BODIPY staining for lipid droplets (Green). DAPI staining for cell nuclei (blue). Bar=10 µm (**p <0.01, ***p <0.001).

We overexpressed *IRX3* by plasmid transfection in the HepG2 cell line. The oleic acid (OA) and palmitic acid (PA)-induced lipid droplet accumulation was significantly decreased in *IRX3*-Flag–positive HepG2 cells (**Figures 5A, B** **)**. The same phenomenon was found in the L02 hepatic cell line (**Figures S1A, B** **)**. Conversely, when it was downregulated, LD accumulation was increased in the HepG2 cells (**Figures 5C, D** **)**. These results showed that *IRX3* regulated hepatic lipid metabolism *in vitro*.

### *IRX3* was related to the expression of mitochondrial thermogenic genes

**Figure 6:**
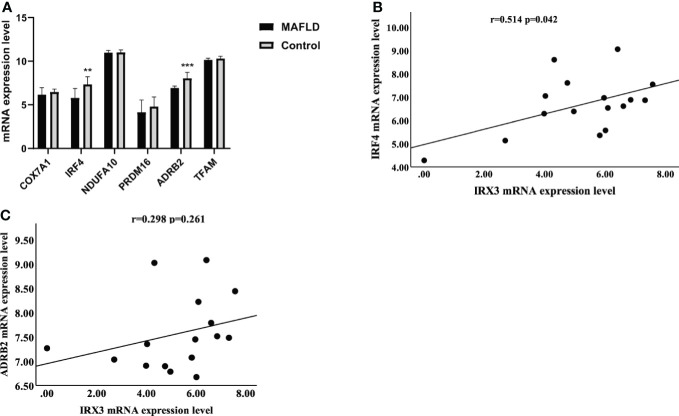
The relationship between *IRX3* and genes related to mitochondrial thermogenesis. **(A)** The mRNA expression levels of thermogenesis-related genes in liver tissues of MAFLD patients and controls (**p <0.01, ***p <0.001). **(B)** Correlation analysis between the expression levels of *IRX3* and *IRF4*. **(C)** Correlation analysis between the expression levels of *IRX3* and *ADRB2*.

Previous studies have shown that *IRX3* affected lipid metabolism by regulating mitochondrial function in adipocytes (36). By screening the RNA-seq results, we found that mitochondrial thermogenesis-related gene expression was decreased in the liver tissue of MAFLD patients (**Figure 6A**). Pearson’s correlation analysis showed that the expression of *IRX3* in liver tissues was positively correlated with that of *IRF4* (**Figure 6B**, r=0.514, *p*=0.042), while there was no relationship between the expression of *IRX3* and *ADRB2* (Adrenoceptor beta 2) (**Figure 6C** r=0.298, *p*=0.261).

### Inhibition of *IRX3* expression induced mitochondrial dysfunction

**Figure 7:**
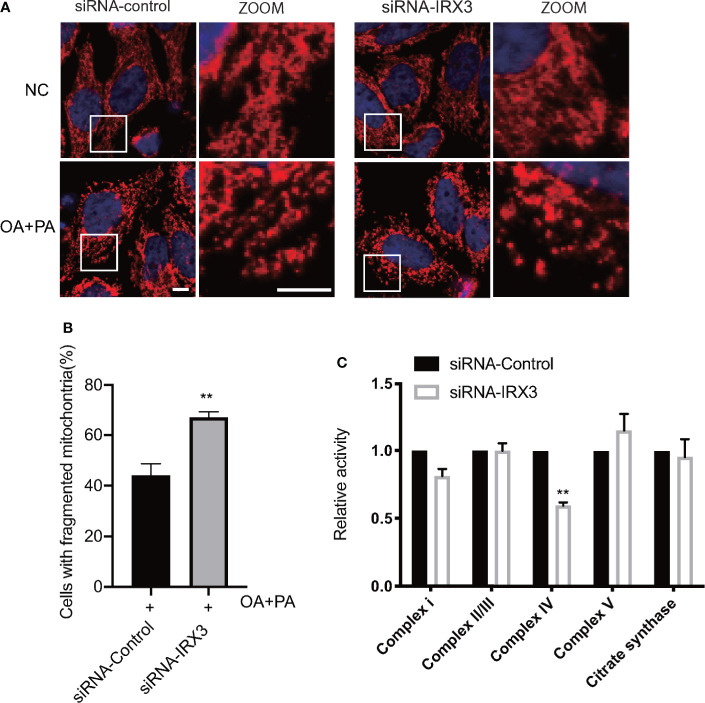
The effects of *IRX3* on mitochondrial function. **(A)** HepG2 cells were transfected with *IRX3* siRNA and control siRNA, mitochondria were stained red by TOM20. Bar=5µm. **(B)** Quantification of the number of cells with fragmented mitochondria. **(C)** The activity of the mitochondrial complexes was detected in cells transfected with *IRX3* siRNA or control. Citrate synthase was used as a mitochondrial control. **p< 0.01.

We changed the expression of *IRX3* in the HepG2 cell line through RNAi or overexpression, TOM20 was used to stain the mitochondria, and overexpression of *IRX3* did not cause a significant change in mitochondrial morphology (**Figure S2A, B** **)**. However, inhibition of *IRX3* expression significantly increased mitochondrial fragmentation (**Figures 7A, B** **)**. Then, we studied the activity of mitochondrial complexes and observed that the activity of mitochondrial complex IV was decreased after inhibition of *IRX3* expression (**Figure 7C**, *p*<0.01). The effect of *IRX3* on mitochondria might be an important mechanism by which *IRX3* regulated hepatic lipid metabolism of MAFLD.

## Discussion

Our study demonstrated that *IRX3* played an important role in regulating hepatic lipid metabolism in MAFLD. The expression of *IRX3* was decreased in liver tissues of patients with MAFLD, and the overexpression of *IRX3* reduced the LD accumulation in L02 and HepG2 cells. Mechanistically, inhibition of *IRX3* led to the increased mitochondrial fragmentation and decreased mitochondrial complex IV activity in HepG2 cells.

Transcriptome- and genome-wide association analyses and epigenetic studies are widely used to reveal the molecular pathological mechanisms of MAFLD. A large number of valuable genes of lipid metabolism have been shown to regulate by several important transcription factors, such as *PPARs*, *FXR*, *C/EBPs*, *SREBPs* and *Zfp423* (37). The transcription factors associated with the MAFLD, including *FXR* agonists and *PPAR-α/δ* agonists, have been studied in late-stage clinical trials to treat NASH (38–40).

In the present study, transcriptome analysis was performed on liver tissues from patients with MAFLD. We found the abnormal expression gene enrichment in pathways related to lipid metabolisms, such as fatty acid metabolic process, monocarboxylic acid metabolic process and pyruvate biosynthetic process. More importantly, it contained the key *PPAR-α* signaling pathway of MAFLD (**Figure 1B** and **Table S3**). These findings suggested that the RNA-Seq data were credible and accurate despite small sample sizes. Previous studies have shown that transcription factors were involved in lipid metabolism, autophagy, endoplasmic reticulum stress, inflammatory response, apoptosis and other biological processes. Transcription factors have also been shown to paly an important role in the occurrence and development of metabolic diseases such as obesity, dyslipidemia, Type 2 diabetes and MAFLD (41, 42). Some of the transcription factors (*MYC*, *IRF4*, and *MAFF*), which were found in differentially expressed gene profiles of the study, were shown to regulate lipid metabolism (30–32). However, little is known about the role of *IRX3* in hepatic lipid metabolism. The present study focused on how *IRX3* played an important role in the pathogenesis of MAFLD by regulating hepatic lipid metabolism.

The *IRX3* gene is a member of the Iroquois homeobox gene family (43). *IRX3* was shown to play an important roles in pancreatic islets regulating the ratio of α and β cells and the expression of ghrelin associated with calorie intake and body composition (33, 44). The association of *IRX3* variants with obesity has been demonstrated. Genetic analysis results indicated that *IRX3* polymorphisms of rs1126960 and rs3751723 were related to obesity (45). It was also observed that there was a relationship between *IRX3*, newborn birth weight and BMI. Genotypes rs8053360 CC and rs1126960 GG were related to body weight and BMI, particularly among female individuals (46), and the polymorphism rs3751723 in *IRX3* has been associated with obesity (47). In the present study, immunohistochemical staining and RNA-seq results from liver tissue showed that the expression of *IRX3* was decreased in MAFLD patients. Consistent with the latest clinical practice guidelines of the Asian Pacific Association for the Study of the Liver (APASL) on MAFLD, which have stated that MAFLD was closely associated with BMI, TC, TG, and LDL-C (48). The expression level of *IRX3* in liver tissue was negatively correlated with serum levels of TC, TG, LDL-C and UA in the study. However, further studies with larger sample sizes are needed to elucidate the correlation between the expression level of *IRX3* and clinical predictors of histopathological severity of MAFLD.

Previous studies have shown that *IRX3* gene expression was controlled by long-range enhancers from the intronic regions of the *FTO* gene (19). *FTO* overexpression damaged hepatocellular mitochondria and inhibited the decomposition of lipids, resulting in lipid accumulation in the liver (49). Chromatin immunoprecipitation sequencing results demonstrated that *IRX3* and *IRX5*, a homologous gene of *IRX3*, regulated mitochondrial gene clusters in early differentiating mouse primary pre-adipocytes from both visceral and subcutaneous white adipocyte depots (50, 51). Mitochondria are known to play a crucial role in lipid metabolism in cells (52–54). In the present study, we found that mitochondrial thermogenesis-related gene expression was decreased in the liver tissue of MAFLD patients, and the expression of *IRX3* in liver tissues was positively correlated with that of *IRF4.* The decreased expression of *IRF4* has been shown to inhibit mitochondrial function and reduce thermogenesis (55, 56). We inhibited the expression of *IRX3* by RNAi led to the increased mitochondrial fragmentation and decreased mitochondrial complex IV activity in HepG2 cells. Mitochondrial complex IV, as an important part of the mitochondrial oxidative respiratory chain, is the regulatory center of mitochondrial oxidative phosphorylation (57). The increased activity of mitochondrial complex IV promoted mitochondrial oxygen consumption rate and accelerated cell energy metabolism (58). Studies have shown that a specific decrease in mitochondrial complex IV activity led to a decrease in cellular fatty acid oxidation and lipid accumulation in adipocytes (59). These results suggested that the regulation of lipid metabolism by *IRX3* might be associated with the transcriptional regulation of mitochondrial-related gene expression. Studies on the effects of other target genes regulated by *IRX3* on fatty acid metabolism will contribute to the understanding of the role of *IRX3* in MAFLD.

## Conclusions

This study explored the regulatory factors associated with MAFLD at the genome level by RNA-seq. More importantly, this study demonstrated that *IRX3* regulated hepatic lipid metabolism, and also revealed the effect of *IRX3* on mitochondria might be an important mechanism by which *IRX3* regulated hepatic lipid metabolism of MAFLD. *IRX3* might be a new therapeutic target for MAFLD.

## Data availability statement

The original contributions presented in the study are publicly available. This data can be found here: NCBI, GSE183229.

## Ethics statement

Written informed consent was obtained from the individual(s) for the publication of any potentially identifiable images or data included in this article.

## Author contributions

YM and GC: design and performance of experiments, data collection and analysis, manuscript preparation; JY, QL, and ZT: conduct of some experiments and data analysis; WF, XL, and ZH: pathologic and statistical analysis; ZS: general guidance, concept development; JL overall supervision, design, data analysis, funding support, and manuscript preparation and finalization. All authors contributed to the article and approved the submitted version.

## Funding

This work was supported by the National Natural Science Foundation of China (Grant No. 82070679), Hunan Province Science and Technology Grant (Grant No. 2019GK5010), Natural Science Foundation of Hunan Province, China (Grant No. 2019JJ50870), and Research Grant of Tianqing for Liver Diseases (TQGB20210181).

## Conflict of interest

The authors declare that the research was conducted in the absence of any commercial or financial relationships that could be construed as a potential conflict of interest.

## Publisher’s note

All claims expressed in this article are solely those of the authors and do not necessarily represent those of their affiliated organizations, or those of the publisher, the editors and the reviewers. Any product that may be evaluated in this article, or claim that may be made by its manufacturer, is not guaranteed or endorsed by the publisher.

## Supplementary material

Supplementary Figure 1 The effect of *IRX3* on lipid droplet accumulation in the L02 cell line. **(A)** L02 cells were transfected with the *IRX3*-Flag (OE *IRX3*, Red) and vector plasmid (NC). **(B)** Quantification of the number of lipid droplets per cell (****p <*0.001).

Supplementary Figure 2 The effects of *IRX3* on mitochondrial morphology. **(A)** HepG2 cells were transfected with the *IRX3*-Flag (OE *IRX3*, white) and vector plasmid (NC), and mitochondria were stained red by TOM20. *IRX3*-Flag-positive cells were marked by white dashed boxes. Bar=10µm. **(B)** Quantification of the number of cells with fragmented mitochondria (*p*>0.05).
