# Supplementary figures and images for: IRX3 plays an important role in the pathogenesis of metabolic-associated fatty liver disease by regulating hepatic lipid metabolism
Source: Front Endocrinol (Lausanne). 2022 Jul 26;13:895593. doi: 10.3389/fendo.2022.895593 (PMC9360787; doi:10.3389/fendo.2022.895593)

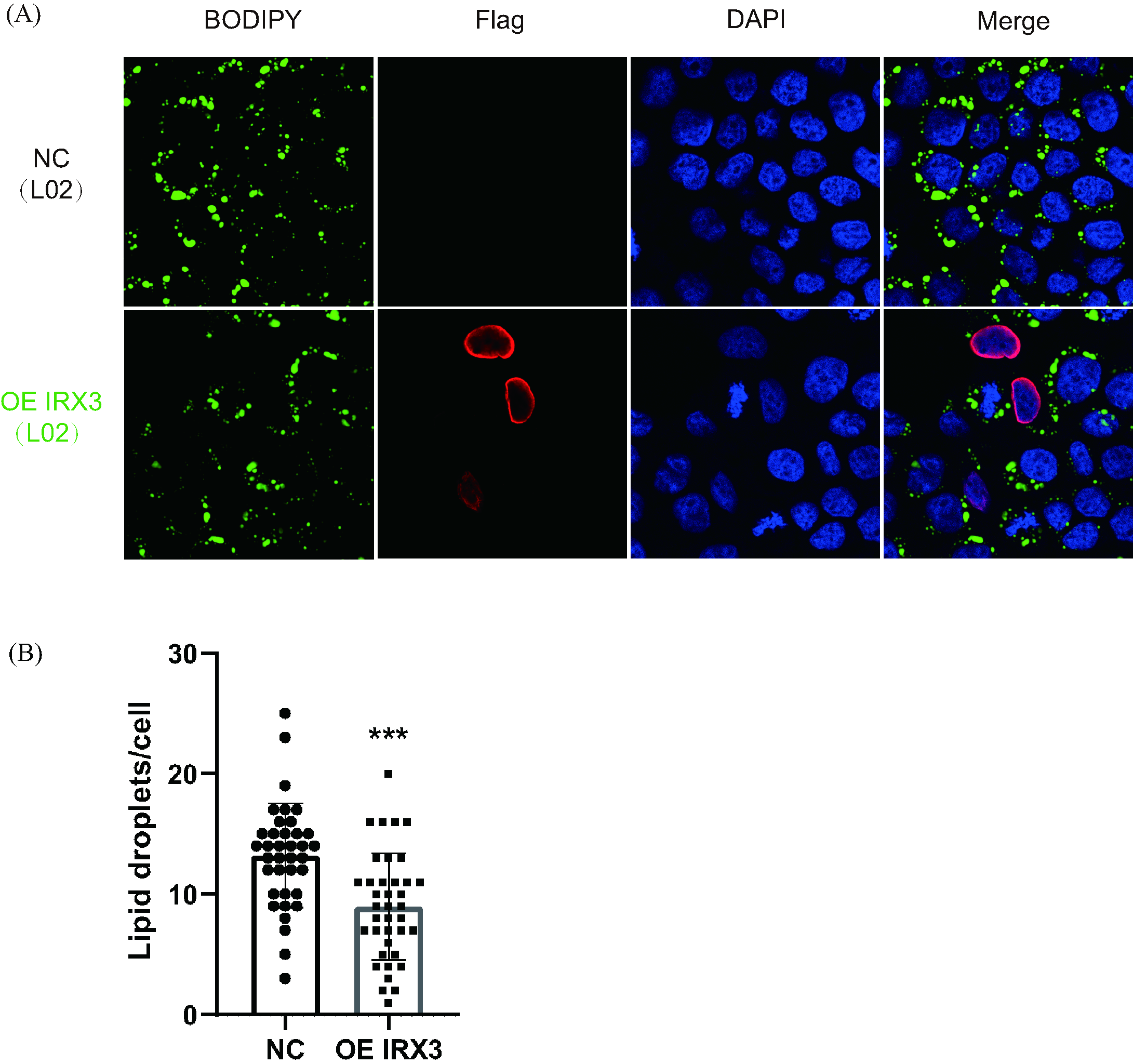

Supplement: Supplementary Figure 1 — The effect of IRX3 on lipid droplet accumulation in the L02 cell line. (A) L02 cells were transfected with the IRX3-Flag (OE IRX3, Red) and vector plasmid (NC). (B) Quantification of the number of lipid droplets per cell (***p <0.001). [file Image_1.tif]

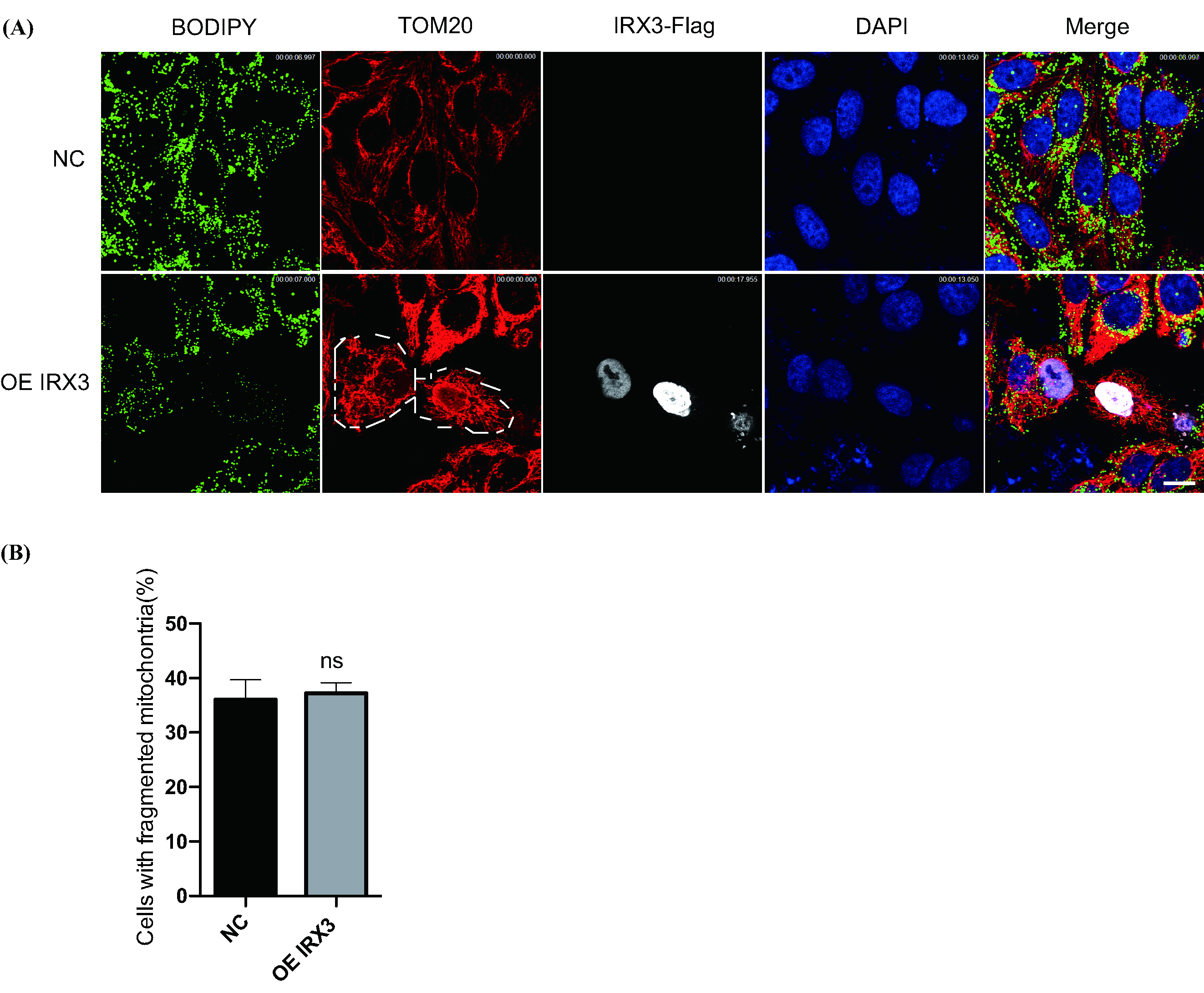

Supplement: Supplementary Figure 2 — The effects of IRX3 on mitochondrial morphology. (A) HepG2 cells were transfected with the IRX3-Flag (OE IRX3, white) and vector plasmid (NC), and mitochondria were stained red by TOM20. IRX3-Flag-positive cells were marked by white dashed boxes. Bar=10µm. (B) Quantification of the number of cells with fragmented mitochondria (p>0.05). [file Image_2.tif]
